# Circulating Dipeptides in Cancer: Degradation Fragments or Functional Metabolites?

**DOI:** 10.3390/ijms27104438

**Published:** 2026-05-15

**Authors:** Kyung-Hee Kim, Byong Chul Yoo

**Affiliations:** 1 Department of Applied Chemistry, School of Science and Technology, Kookmin University, Seoul 02707, Republic of Korea; kyungheekim@kookmin.ac.kr; 2 Antibody Research Institute, Kookmin University, Seoul 02707, Republic of Korea; 3 Diagnostic Research Team, InnoBation Bio R&D Center, Seoul 03929, Republic of Korea

**Keywords:** cancer metabolomics, circulating dipeptides, peptide metabolism, tumor metabolism, proteolysis, bioactive peptides, metabolic communication

## Abstract

Advances in mass spectrometry-based metabolomics have enabled the detection of numerous small molecules in biological systems, revealing complex metabolic alterations associated with cancer. Among these, dipeptides are consistently detected in plasma, serum, and tumor tissue metabolomic profiles, yet their biological significance is not fully understood. In most studies, circulating dipeptides are interpreted as nonspecific byproducts of protein degradation generated during increased proteolysis. However, accumulating evidence suggests that at least some endogenous dipeptides may have biological activities, including antioxidant effects, metabolic modulation, and potential signaling functions. In this review, we examine the possible origins, transport mechanisms, and biological implications of circulating dipeptides in cancer metabolomics. We discuss multiple sources of dipeptide generation, including intracellular proteolysis, autophagy, extracellular matrix remodeling, tumor cell death, host tissue catabolism, and microbiome metabolism. We also summarize current knowledge regarding peptide transport systems and intracellular dipeptide metabolism that may regulate the fate of these molecules within mammalian systems. In addition, evidence supporting the biological activities of certain endogenous dipeptides is reviewed to evaluate the possibility that some circulating dipeptides may function as bioactive metabolites. Finally, we propose conceptual frameworks for interpreting circulating dipeptides in cancer, including their potential roles as indicators of protein turnover, intermediates in amino acid recycling, stress-buffering molecules, metabolic signals, or components of tumor–host metabolic communication. A better understanding of circulating dipeptides may provide new insights into cancer metabolism and reveal previously overlooked metabolite classes with potential biomarker or functional significance.

## 1. Introduction

Advances in mass spectrometry-based metabolomics have enabled the comprehensive profiling of small molecules in biological systems, revealing complex metabolic signatures associated with cancer development and progression [1,2]. Recent large-scale metabolomic studies and integrative cancer profiling efforts have further expanded the identification of circulating metabolites associated with tumor biology and systemic metabolic reprogramming [3,4].

Among the diverse metabolite classes detected in cancer metabolomic studies, dipeptides have emerged as a recurrent yet relatively underexplored component of metabolic profiles. Several metabolomic analyses have reported the presence of dipeptides in plasma, serum, and tumor tissues, often as part of broader metabolite signatures rather than primary analytes [5,6,7].

In this review, the term “circulating dipeptides” is primarily used to refer to dipeptides detected in systemic biofluids, particularly plasma and serum. Dipeptides identified in tumor tissues are discussed in the context of their potential contribution to circulating pools, whereas urinary peptides are not considered a primary focus unless explicitly indicated.

In this context, it is important to clarify the use of the term “metabolomics.” Although dipeptides are structurally peptides, they are frequently detected in mass spectrometry-based metabolomic platforms due to their small size and physicochemical properties, which overlap with those of conventional metabolites. As a result, dipeptides are often included in untargeted metabolomic datasets alongside amino acids, lipids, and other small molecules.

Conceptually, this review considers dipeptides at the interface between metabolomics and peptidomics. While larger peptides are typically studied within proteomic or peptidomic frameworks, short peptides such as dipeptides can function as metabolic intermediates and are therefore commonly analyzed and interpreted within metabolomic studies.

Despite their frequent detection, the biological significance of circulating dipeptides remains unclear. In many metabolomic analyses, they are interpreted as nonspecific byproducts of protein degradation generated through proteolytic processes within tissues or during systemic metabolic turnover [8,9,10,11,12], and are therefore often categorized as metabolic noise or degradation fragments without further mechanistic interpretation.

However, this view may be overly simplistic. Emerging evidence suggests that some small peptides, including certain endogenous dipeptides, can possess biological activities [13,14,15]. In addition, specific transport systems capable of mediating the uptake of dipeptides, such as proton-coupled oligopeptide transporters, indicate that dipeptides are not merely transient degradation products but may represent physiologically relevant metabolic intermediates [16,17].

In the context of cancer, several metabolomic studies have reported altered metabolic profiles associated with tumor burden, metabolic reprogramming, or treatment response, in which dipeptides are detected as part of broader metabolite signatures rather than as primary analytes [18,19]. These observations raise an important question: are circulating dipeptides simply passive reflections of increased proteolysis in cancer, or could they represent previously underappreciated functional metabolites that participate in tumor–host metabolic interactions?

This question is particularly relevant given the profound metabolic remodeling that occurs in cancer. Tumor cells undergo extensive alterations in nutrient utilization, protein turnover, and amino acid metabolism in order to sustain rapid proliferation and adapt to the tumor microenvironment [20,21,22]. Such metabolic changes could potentially influence the generation, transport, and systemic circulation of small peptides derived from intracellular or extracellular proteolysis.

Moreover, the tumor microenvironment is characterized by elevated protease activity, enhanced autophagy, and increased protein turnover, all of which may contribute to the production of short peptide fragments [23,24,25]. These processes could generate pools of dipeptides that enter the circulation, thereby becoming detectable in metabolomic analyses of patient plasma or serum samples.

At the same time, accumulating evidence indicates that certain dipeptides possess biological activities that extend beyond simple metabolic intermediates. Histidine-containing dipeptides such as carnosine and anserine have been shown to exhibit antioxidant properties, modulate cellular metabolism, and influence signaling pathways relevant to cancer biology [13,26,27]. These observations raise the possibility that at least a subset of circulating dipeptides may function as bioactive molecules with regulatory roles in tumor biology.

Despite these intriguing observations, the field lacks a comprehensive framework for understanding the origin, metabolism, and potential functional roles of circulating dipeptides in cancer. Most metabolomic studies report the presence of dipeptides as isolated findings, without integrating them into broader models of tumor metabolism or tumor–host metabolic interactions.

In this review, we examine the emerging evidence regarding circulating dipeptides detected in cancer metabolomics studies and explore the possible biological origins and functional implications of these molecules. We first discuss the potential sources of dipeptides in cancer, including intracellular proteolysis, autophagy, and extracellular protease activity. We then summarize current knowledge regarding dipeptide transport and metabolism in mammalian systems. Finally, we consider whether circulating dipeptides might represent functional metabolites involved in metabolic signaling, immune regulation, or tumor–host communication, rather than merely degradation fragments.

**Figure 1:**
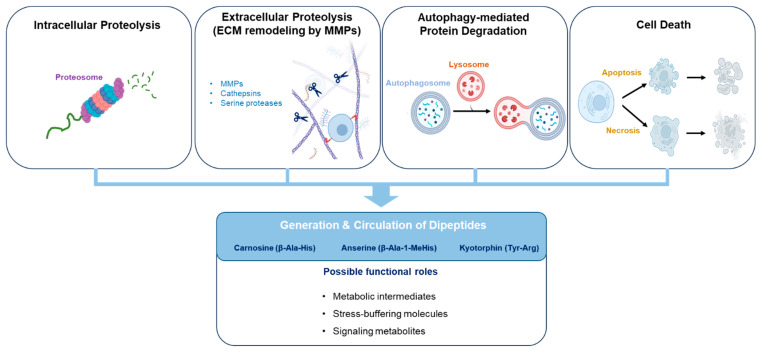
Conceptual framework of circulating dipeptides in cancer. Circulating dipeptides may arise from multiple sources, including intracellular proteolysis, extracellular matrix degradation, and tissue turnover. These dipeptides can enter the circulation and may reflect systemic metabolic alterations associated with cancer. Several potential roles are illustrated, including their function as metabolic byproducts, indicators of proteolytic activity, and potential contributors to metabolic communication, with representative examples such as carnosine, anserine, and kyotorphin. The figure summarizes the relationships among peptide generation, circulation, and their potential biological relevance in cancer. Abbreviations: ECM, extracellular matrix; MMPs, matrix metalloproteinases. Created with BioRender.com.

By integrating insights from metabolomics, protein turnover, and peptide biology, this review aims to provide a conceptual framework for interpreting the presence of circulating dipeptides in cancer and to highlight key knowledge gaps that warrant further investigation (Figure 1).

For clarity, several key terms used throughout this review are defined as follows. “Functional metabolites” refers to metabolites that exert measurable biological effects beyond their role as intermediates in metabolic pathways, such as influencing cellular processes including redox balance, enzyme activity, or signaling pathways. Here, “metabolic signals” refers to metabolites that are capable of modulating cellular communication, either directly or indirectly, such as through interactions with receptors, transporters, or intracellular signaling pathways. By contrast, “metabolic communication” is used in a broader sense to describe the exchange of metabolites between cells or tissues, which can contribute to coordinated metabolic responses across the organism, including interactions between tumor and host tissues.

## 2. Sources of Circulating Dipeptides in Cancer

While these processes are introduced in the Introduction as general concepts, the following sections provide a more detailed mechanistic breakdown of the major sources contributing to circulating dipeptides. The detection of dipeptides in circulating metabolomic profiles raises an important question regarding their biological origin. In cancer patients, multiple processes associated with tumor growth, metabolic remodeling, and systemic host responses may contribute to the generation and release of small peptide fragments into the circulation. Although the precise sources of circulating dipeptides remain incompletely understood, several mechanisms have been proposed, including intracellular protein degradation, autophagy-mediated proteolysis, extracellular protease activity, and tissue remodeling within the tumor microenvironment [20,23,24].

Understanding the potential origins of circulating dipeptides is essential for interpreting metabolomic findings and distinguishing between passive degradation products and metabolites that may possess functional relevance in tumor biology.

### 2.1. Intracellular Proteolysis and Protein Turnover

Intracellular protein turnover represents a major source of peptide fragments in mammalian cells. In rapidly proliferating tumor cells, protein synthesis and degradation are both markedly elevated in order to maintain cellular homeostasis and support metabolic adaptation [21,28]. As a consequence, enhanced proteolytic activity within cancer cells may generate increased quantities of small peptides that can subsequently enter the cytosolic metabolite pool. Intracellular protein degradation is primarily mediated by the ubiquitin–proteasome system and lysosomal pathways, including autophagy [29].

One of the principal pathways responsible for intracellular protein degradation is the ubiquitin–proteasome system. In this pathway, proteins destined for degradation are tagged with ubiquitin molecules and subsequently processed by the proteasome complex, which cleaves proteins into short peptide fragments [29,30]. While proteasomal degradation typically produces peptides ranging from several to tens of amino acids in length, further processing by cytosolic peptidases can generate smaller peptides, including dipeptides and free amino acids [31,32]. The proteasome exhibits cleavage preferences influenced by amino acid sequence context, frequently generating peptides with hydrophobic or basic C-terminal residues, which are subsequently processed by cytosolic peptidases.

Cancer cells frequently exhibit increased proteasome activity as part of their adaptation to high rates of protein synthesis and cellular stress. This elevated proteolytic flux could potentially contribute to the accumulation of small peptides within tumor cells, some of which may diffuse or be transported into extracellular spaces and ultimately enter the circulation [28,33,34].

### 2.2. Autophagy and Lysosomal Proteolysis

Autophagy represents another major mechanism contributing to intracellular protein degradation in cancer. Through this process, cytoplasmic components—including proteins, organelles, and protein aggregates—are engulfed within autophagosomes and delivered to lysosomes for degradation [25].

Within lysosomes, proteins are subjected to proteolytic cleavage by cathepsins and other lysosomal proteases, generating a mixture of amino acids and short peptides. These lysosomal proteases generate peptide fragments with diverse sequence characteristics, which may undergo further trimming by exopeptidases. While the majority of these degradation products are recycled for cellular metabolism, partial proteolysis may generate small peptides, including dipeptides [35].

Autophagy is frequently upregulated in tumors, particularly under conditions of metabolic stress such as hypoxia or nutrient deprivation [23]. In these contexts, lysosomal proteolysis may contribute substantially to the intracellular generation of peptide fragments that can potentially be exported from tumor cells or released following cell turnover.

### 2.3. Extracellular Proteolysis in the Tumor Microenvironment

In addition to intracellular processes, proteolytic activity in the extracellular space of the tumor microenvironment may also contribute to the generation of circulating dipeptides. Tumor progression is frequently associated with extensive remodeling of the extracellular matrix, driven by various proteases, including matrix metalloproteinases (MMPs), cathepsins, and serine proteases [24,36].

These enzymes break down extracellular matrix proteins and other structural components, producing a range of peptide fragments that can accumulate in the local microenvironment. Subsequent enzymatic processing by exopeptidases and other peptidases can further shorten these fragments, potentially producing dipeptides capable of entering the circulation [35,37].

Because many tumors exhibit elevated protease activity associated with invasion and metastasis, extracellular proteolysis may represent an important contributor to systemic pools of peptide fragments detected in cancer metabolomic studies.

### 2.4. Tumor Cell Death and Tissue Remodeling

Another potential source of circulating dipeptides arises from tumor cell death and tissue remodeling processes. Both apoptosis and necrosis can release intracellular proteins into the extracellular environment, where they become substrates for proteolytic degradation [38,39]. Apoptosis is characterized by regulated proteolysis mediated by caspases, resulting in controlled cleavage of intracellular proteins into defined fragments. In contrast, necrosis involves loss of membrane integrity and uncontrolled release of cellular contents, followed by extracellular proteolysis of released proteins by proteases in the tissue microenvironment.

In addition, cancer therapies such as chemotherapy, radiotherapy, and targeted treatments often induce significant levels of tumor cell death, which may further increase the release of protein fragments and peptides into circulation. Proteolytic processing of these released proteins by circulating or tissue proteases could generate dipeptides that become detectable in plasma metabolomic profiles [40].

Beyond tumor cells themselves, systemic host responses to cancer—including inflammation, muscle wasting, and metabolic stress—may also contribute to increased protein turnover and peptide generation in peripheral tissues. These processes may collectively influence the composition of circulating peptide metabolites in cancer patients.

### 2.5. Contributions from the Microbiome

The gut microbiome represents an additional potential contributor to circulating dipeptide pools. Intestinal bacteria possess extensive proteolytic capabilities and can generate a wide range of small peptides through the degradation of dietary and host-derived proteins [41,42].

Some of these microbial peptides may be absorbed through intestinal peptide transport systems such as PEPT1 and enter systemic circulation, thereby contributing to the metabolomic signatures detected in plasma samples. Given the growing evidence linking the microbiome to cancer metabolism and immune regulation, microbial production of dipeptides could represent an underappreciated component of circulating peptide metabolite profiles in cancer patients [43,44].

### 2.6. Interpreting Circulating Dipeptides: Degradation Products or Functional Metabolites?

Taken together, multiple biological processes may contribute to the presence of dipeptides in circulation. In addition to proteolytic mechanisms, certain dipeptides can also be generated through specific enzymatic synthesis. For example, histidine-containing dipeptides such as carnosine are synthesized by dedicated enzymes (e.g., carnosine synthase) from their constituent amino acids. These biosynthetic pathways produce functionally relevant dipeptides with defined biological roles, indicating that not all circulating dipeptides arise solely from protein degradation. Accordingly, circulating dipeptide pools may reflect a combination of degradative and biosynthetic processes. Increased proteolysis within tumor cells, enhanced autophagy, extracellular matrix remodeling, tumor cell death, and microbial metabolism may all generate small peptide fragments that become detectable through metabolomic analysis.

These diverse origins complicate the interpretation of circulating dipeptides. The major biological sources contributing to circulating dipeptide generation in cancer are summarized in Table 1. On the one hand, their presence may simply reflect elevated protein turnover associated with tumor growth and tissue remodeling. On the other hand, the repeated detection of specific dipeptides across independent metabolomic studies raises the possibility that at least a subset of circulating dipeptides may possess functional biological roles.

Distinguishing between these possibilities requires a deeper understanding of dipeptide transport, metabolism, and potential biological activities within mammalian systems. In the following sections, we therefore examine the mechanisms by which dipeptides can be transported and metabolized, and consider whether these molecules might participate in previously unrecognized aspects of cancer metabolism and tumor–host communication.

However, most of these proposed sources are inferred from general biological processes rather than directly demonstrated in the context of circulating dipeptides. As a result, the relative contribution of each mechanism to systemic dipeptide pools remains unclear, and experimental evidence distinguishing tumor-derived from host-derived peptides is currently limited. Importantly, these processes differ not only in their contribution to peptide generation but also in the types of peptide fragments they produce. For example, proteasomal degradation predominantly generates short peptides through ATP-dependent cleavage, whereas lysosomal proteolysis and extracellular matrix degradation involve distinct protease systems that may produce peptides with different sequence characteristics and stability. These mechanistic differences may influence the composition of circulating dipeptide profiles observed in cancer metabolomics.

## 3. Transport and Metabolism of Dipeptides

Although dipeptides are frequently detected in metabolomic analyses of biological samples, their physiological handling within mammalian systems remains only partially understood. A key factor that determines whether circulating dipeptides represent transient degradation products or biologically relevant metabolites is the existence of dedicated transport and metabolic pathways capable of regulating their cellular uptake and utilization.

Evidence accumulated over the past several decades indicates that mammalian cells possess specialized mechanisms for the transport and metabolism of small peptides [16,17,47]. These systems suggest that dipeptides may participate in metabolic processes that extend beyond simple protein degradation.

However, many of the studies reporting circulating dipeptides rely on untargeted metabolomic approaches, and the distinction between biologically generated peptides and those arising from sample processing or degradation remains incompletely resolved. This limitation complicates the interpretation of dipeptide origin and biological significance.

### 3.1. Proton-Coupled Oligopeptide Transporters

Cellular uptake of dipeptides and tripeptides is primarily mediated by proton-coupled oligopeptide transporters of the solute carrier family 15 (SLC15) [47]. Among these, PEPT1 (SLC15A1) and PEPT2 (SLC15A2) are the most extensively characterized.

PEPT1 is mainly expressed in the small intestine, where it plays a central role in the absorption of dietary peptides generated during protein digestion [16,17]. This process is driven by the transmembrane proton gradient, allowing efficient uptake of a wide range of dipeptides and tripeptides into intestinal epithelial cells.

In contrast, PEPT2 has a broader tissue distribution and is found in organs such as the kidney, brain, lung, and various immune cell types [47]. This transporter is characterized by higher affinity but lower transport capacity compared with PEPT1 and is thought to contribute to the systemic handling and cellular uptake of circulating peptide metabolites.

Importantly, these transporters display remarkably broad substrate specificity. A wide range of dipeptides composed of different amino acid combinations can be transported through PEPT-mediated mechanisms, indicating that dipeptides can be actively transported into cells and incorporated into cellular metabolic pathways [16,48].

These transporters function as proton-coupled symporters that mediate the influx of dipeptides and tripeptides into cells by utilizing transmembrane proton gradients [11,41]. Their primary physiological role is therefore associated with peptide uptake rather than secretion. Accordingly, there is currently limited evidence supporting a direct role of PEPT1 or PEPT2 in the export of dipeptides into the circulation.

In the intestine and kidney, PEPT1 and PEPT2 are known to contribute to dietary peptide absorption and renal peptide reuptake, respectively, thereby influencing the systemic availability of peptide-derived metabolites [16,17,47]. While these transporters may indirectly affect circulating dipeptide levels through uptake and redistribution processes, their involvement in regulating peptide release into the bloodstream remains unclear.

Although PEPT transporters exhibit broad substrate specificity, they are not entirely non-selective. Substrate recognition is influenced by peptide length, charge, and amino acid composition, with certain dipeptides showing higher transport efficiency than others [16,48]. This suggests that circulating dipeptide profiles may be shaped not only by proteolytic processes but also by transporter-mediated selectivity. Mechanistically, this process involves proton-coupled symport in which dipeptide uptake is driven by transmembrane proton gradients, allowing efficient intracellular accumulation of peptide substrates even under low extracellular concentrations.

**Figure 2:**
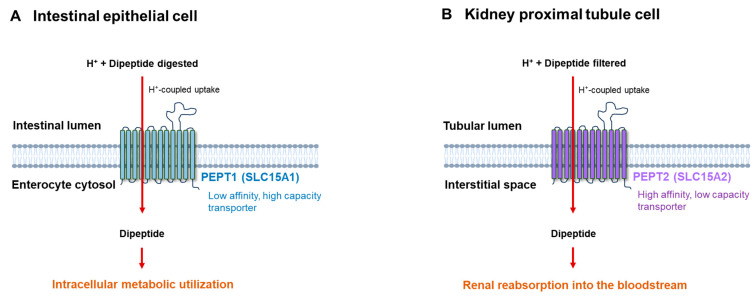
Schematic representation of PEPT1- and PEPT2-mediated dipeptide transport in intestinal and renal epithelial cells. (**A**) PEPT1 (SLC15A1), expressed in intestinal epithelial cells, functions as a low-affinity, high-capacity transporter that mediates proton-coupled uptake of dipeptides from the intestinal lumen into the cytosol, where they contribute to intracellular metabolic processes. (**B**) PEPT2 (SLC15A2), expressed in renal proximal tubule cells, functions as a high-affinity, low-capacity transporter involved in the reabsorption of filtered dipeptides from the tubular lumen. Both transporters operate as H^+^-coupled symporters and primarily mediate peptide influx rather than secretion, thereby contributing to the systemic handling of peptide-derived metabolites. Created using Microsoft PowerPoint.

PEPT1 is primarily involved in intestinal absorption and PEPT2 in renal reabsorption (Figure 2) [16,47,49]. In addition to classical dipeptide transporters, recent studies have suggested that multiple carrier systems, including LAT family transporters, may contribute to the handling of ultrashort peptides under specific physiological conditions, highlighting a more complex and potentially context-dependent peptide transport network [49]. This transport mechanism is driven by transmembrane proton gradients, enabling the intracellular accumulation of dipeptides even under low extracellular concentrations and thereby facilitating efficient peptide utilization within cells.

In addition to PEPT1 and PEPT2, other transport systems capable of handling dipeptides have been reported, although their physiological relevance for systemic dipeptide transport appears to be more limited. For example, certain peptide transport activity has been associated with PHT family transporters (SLC15A3 and SLC15A4), which are primarily localized to intracellular compartments and are thought to be involved in immune-related processes rather than bulk peptide absorption [47]. In addition, some amino acid transporters and non-specific uptake mechanisms may contribute indirectly to the handling of small peptides under specific conditions. However, compared with PEPT1 and PEPT2, these systems are less well characterized in the context of circulating dipeptide regulation.

In addition, most functional insights into peptide transport are derived from physiological or pharmacological studies rather than cancer-specific models. Therefore, the extent to which these transport systems contribute to circulating dipeptide dynamics in cancer remains to be fully established. Mechanistically, this limitation highlights a critical gap in our understanding, as transporter-mediated selectivity may influence not only peptide uptake but also the composition of intracellular and potentially circulating dipeptide pools.

### 3.2. Dipeptide Transport in Cancer Cells

PEPT1 and PEPT2 are members of the SLC15 family of proton-coupled oligopeptide transporters and are widely expressed in various tissues [47,50]. While these transporters are well characterized in physiological contexts such as intestinal peptide absorption, their roles in cancer cells remain less clearly defined.

The presence of peptide transport systems in normal tissues raises the possibility that similar mechanisms could be relevant in tumor contexts. However, direct evidence supporting functional dipeptide transport in cancer cells remains limited.

Tumor cells often experience nutrient limitation due to inadequate vascularization and competition within the tumor microenvironment. Under such conditions, the ability to import small peptides could provide a metabolic advantage by supplying amino acids that support biosynthesis and energy metabolism [20,21].

In addition, peptide transporters have attracted considerable interest in oncology because they can facilitate the uptake of peptide-like drugs and prodrugs designed to exploit PEPT-mediated transport pathways [17,47]. These observations support the broader relevance of peptide transport systems, although their functional roles in tumor metabolism remain to be fully established.

Although the extent to which tumor cells utilize dipeptides as metabolic substrates remains poorly characterized, the presence of peptide transport systems suggests that circulating dipeptides could potentially participate in tumor metabolic networks as alternative sources of amino acids.

### 3.3. Intracellular Metabolism of Dipeptides

Once transported into cells, dipeptides can be rapidly hydrolyzed by intracellular peptidases to generate free amino acids. Numerous cytosolic and mitochondrial peptidases capable of cleaving dipeptides have been identified in mammalian cells [10,31]. This hydrolysis is mediated by cytosolic and mitochondrial peptidases, which cleave peptide bonds to release free amino acids that subsequently enter central metabolic pathways. These enzymes contribute to the recycling of peptide fragments generated during intracellular protein turnover.

The resulting amino acids can subsequently enter central metabolic pathways, including protein synthesis, nucleotide biosynthesis, and energy metabolism. In this context, dipeptides may function as intermediates that facilitate amino acid delivery to metabolically active cells.

However, it is also possible that not all dipeptides are immediately degraded following cellular uptake. Some dipeptides may exhibit relative stability or interact with specific cellular targets, allowing them to exert biological effects before enzymatic hydrolysis occurs. Evidence supporting this possibility comes from studies demonstrating biological activity for certain endogenous dipeptides, including histidine-containing dipeptides such as carnosine [15]. In addition, histidine-containing dipeptides such as carnosine, anserine, and homocarnosine have been implicated in metabolic and neurological disorders related to altered dipeptide metabolism, further highlighting their physiological relevance beyond general peptide turnover [51].

These observations suggest that the metabolic fate of dipeptides may vary depending on their sequence composition, cellular context, and the activity of intracellular peptidases.

### 3.4. Stability and Circulation of Dipeptides

For dipeptides to appear in measurable concentrations in circulating metabolomic profiles, they must exhibit at least transient stability within the extracellular environment. Plasma contains multiple peptidases capable of degrading small peptides; however, several metabolomic studies have reported the detection of specific dipeptides in plasma samples from both healthy individuals and cancer patients [5,6,7]. This apparent persistence suggests that at least a subset of circulating dipeptides may be protected from rapid degradation or continuously regenerated, allowing their reproducible detection in plasma metabolomic studies. In addition, chemical modifications of amino acid residues, such as methylation or phosphorylation, may influence peptide stability and susceptibility to proteolytic degradation. Although direct evidence for modified circulating dipeptides remains limited, such modifications could contribute to the persistence and variability of dipeptides detected in metabolomic analyses.

#### 3.4.1. Stability and Reproducibility of Circulating Dipeptides

The persistence of these molecules in circulation may be influenced by multiple factors, including protection from rapid degradation, continuous generation through proteolytic processes, or potential transport across cellular membranes.

Advances in high-resolution metabolomics have revealed that many circulating dipeptides occur reproducibly across independent cohorts and analytical platforms. This reproducibility suggests that at least some circulating dipeptides represent stable metabolic entities rather than stochastic degradation artifacts [52,53].

#### 3.4.2. Mechanisms of Dipeptide Release and Entry into Circulation

The mechanisms by which dipeptides enter the systemic circulation remain incompletely understood and are likely multifactorial. One potential source is the extracellular release of peptides generated through intracellular proteolysis, followed by leakage or passive diffusion across the plasma membrane [8,9,10,11]. In addition, proteolytic activity in the extracellular environment, including tumor-associated proteases and circulating peptidases, may directly generate dipeptides outside cells [20,21,22,23,24,25].

Transporter-mediated processes may also contribute indirectly. While PEPT1 and PEPT2 are primarily involved in cellular uptake rather than export, they can influence systemic peptide availability through intestinal absorption and renal reabsorption, respectively [16,47]. At present, there is limited evidence for dedicated peptide exporters that directly mediate dipeptide secretion into the bloodstream.

### 3.5. Implications for Cancer Metabolism

Taken together, the existence of dedicated peptide transporters and intracellular dipeptide-processing enzymes indicates that mammalian systems are equipped to handle small peptides in a regulated manner. These mechanisms raise the possibility that circulating dipeptides could contribute to metabolic interactions between tumors and the host.

In the context of cancer, where protein turnover, protease activity, and metabolic remodeling are markedly altered, the generation and circulation of dipeptides may reflect dynamic processes occurring within both tumor tissues and systemic host metabolism. Whether these molecules merely represent metabolic byproducts or instead participate in signaling and metabolic regulation remains an open question.

In the following section, we therefore examine emerging evidence suggesting that certain dipeptides may possess biological activities relevant to cancer biology, including effects on cellular metabolism, oxidative stress, and immune regulation.

## 4. Bioactive Dipeptides and Their Potential Roles in Cancer Biology

Although many circulating dipeptides are likely generated through proteolytic degradation, emerging evidence suggests that some dipeptides may persist as relatively stable metabolic entities rather than purely stochastic degradation artifacts. This does not exclude a degradative origin; rather, it indicates that certain dipeptides may exhibit reproducible abundance patterns across samples and conditions. In particular, specific classes of dipeptides, such as histidine-containing dipeptides (e.g., carnosine), have been shown to possess defined biological functions, including antioxidant activity and modulation of cellular metabolism, supporting the notion that at least a subset of dipeptides may have functional relevance beyond passive degradation products [15,52,53].

Among the most extensively studied bioactive dipeptides are histidine-containing dipeptides, which have been shown to exert diverse biochemical and physiological effects. Representative examples of endogenous dipeptides with reported biological activities are summarized in Table 2. To provide a clearer mechanistic overview, we further summarize the proposed functional roles of dipeptides together with their underlying mechanisms and supporting evidence in Table 3. While Table 2 summarizes representative dipeptides identified in metabolomic studies and their reported properties, Table 3 provides a structured overview of their proposed functional roles and underlying mechanisms.

### 4.1. Histidine-Containing Dipeptides

Histidine-containing dipeptides represent one of the best-characterized classes of endogenous dipeptides in vertebrate tissues. The most prominent members of this group include carnosine (β-alanyl-L-histidine), anserine (β-alanyl-N-methylhistidine), and homocarnosine (γ-aminobutyryl-L-histidine) [15,51]. These molecules are widely distributed in excitable tissues such as skeletal muscle and brain but can also be detected in circulating biological fluids.

One of the key biochemical properties of histidine-containing dipeptides is their ability to act as proton buffers and antioxidants. Through the imidazole ring of the histidine residue, these molecules can neutralize reactive oxygen species and reactive carbonyl compounds generated during oxidative stress [26,58]. This property is particularly relevant in cancer, where elevated oxidative stress and disrupted redox balance are common features of tumor metabolism.

Carnosine, in particular, has drawn attention for its potential anti-tumor effects. Several experimental studies suggest that it can suppress the proliferation of certain cancer cell lines and affect glycolytic metabolism [27,59]. Although the underlying mechanisms are not yet fully defined, proposed explanations include effects on cellular energy metabolism, possible suppression of mitochondrial function, and interference with glycolytic enzyme activity.

### 4.2. Dipeptides as Antioxidant and Carbonyl-Scavenging Molecules

In addition to histidine-containing dipeptides, other small peptides have been shown to exhibit antioxidant properties. Short peptide sequences can act as scavengers of reactive oxygen species or reactive carbonyl compounds generated during metabolic stress [60,61,62,63]. Representative examples of dipeptides with antioxidant and carbonyl-scavenging properties include histidine-containing dipeptides such as carnosine (β-Ala–His) and anserine (β-Ala–1-methylhistidine), as well as Gly–His and Ala–His. These dipeptides exhibit enhanced reactivity toward reactive oxygen species and carbonyl compounds, largely due to the presence of histidine residues, which provide imidazole functional groups capable of metal chelation and nucleophilic scavenging reactions [15,56].

In cancer, oxidative stress arises from multiple sources, including mitochondrial dysfunction, oncogenic signaling, and inflammatory processes within the tumor microenvironment. The ability of certain dipeptides to neutralize reactive molecules may therefore influence cellular responses to oxidative damage and metabolic stress.

Furthermore, reactive carbonyl species produced during lipid peroxidation or glucose metabolism can modify proteins and nucleic acids, contributing to cellular dysfunction. Dipeptides capable of reacting with these compounds may serve as protective molecules that buffer metabolic stress within cells and tissues.

Although most studies examining these properties have focused on dietary or synthetic peptides, the presence of endogenous dipeptides with similar biochemical characteristics suggests that naturally occurring peptide metabolites may also participate in cellular defense mechanisms.

### 4.3. Potential Metabolic Regulatory Functions

Beyond their antioxidant properties, some dipeptides may influence cellular metabolism through interactions with metabolic enzymes or signaling pathways. Experimental studies have suggested that certain peptides can modulate glycolysis, mitochondrial respiration, or amino acid metabolism [27]. Proposed mechanisms include direct interaction with glycolytic intermediates or enzymes, as well as indirect modulation of cellular redox state, which can influence metabolic flux.

For example, carnosine has been reported to suppress glycolytic flux in tumor cells [64,65]. This observation is particularly intriguing given the reliance of many cancer cells on aerobic glycolysis for energy production and biosynthetic precursor generation.

If specific dipeptides can influence central metabolic pathways, their presence within the cellular metabolite pool could contribute to metabolic regulation in ways that are not yet fully appreciated. These effects may be particularly relevant in cancer, where metabolic reprogramming is a key feature of tumor cells and could be influenced, at least in part, by small peptide metabolites. This perspective points to a potential link between protein turnover, peptide metabolism, and broader metabolic regulation within cells.

### 4.4. Dipeptides as Potential Signaling Molecules

Another possibility is that certain dipeptides may function as signaling molecules that influence cellular communication. This could involve interactions with receptors, modulation of transporter activity, or indirect effects on intracellular signaling pathways, although such mechanisms remain largely hypothetical for most endogenous dipeptides.In biological systems, small peptides are often known to act as signaling mediators, hormones, or neuromodulators [10,66]. While most well-characterized signaling peptides are longer than dipeptides, the idea that short peptides may also interact with receptors or transporters continues to be explored. For example, the dipeptide kyotorphin (Tyr–Arg) has been reported to function as a neuroactive signaling molecule, supporting the possibility that short peptides can participate in receptor-mediated or signaling processes. In addition, small peptides have been shown in some contexts to modulate receptor activity or intracellular signaling pathways, although such mechanisms remain largely unexplored for most endogenous dipeptides.

In the context of cancer, signaling molecules derived from metabolic pathways can influence immune responses, tumor growth, and interactions between tumor cells and the surrounding microenvironment. If specific dipeptides are capable of modulating signaling pathways or cellular communication networks, their presence in circulation could reflect previously unrecognized aspects of tumor–host metabolic interactions.

At present, however, the signaling roles of endogenous dipeptides remain poorly characterized. Further studies will be required to determine whether circulating dipeptides possess receptor-mediated activities or influence signaling networks within cancer tissues.

Taken together, while these observations raise the possibility that certain dipeptides may participate in signaling processes, their roles as endogenous signaling molecules remain incompletely understood and require further experimental validation.

### 4.5. Functional Versus Incidental Peptide Metabolites

The observations discussed above highlight a critical conceptual challenge in interpreting circulating dipeptides detected in metabolomic studies. On the one hand, the generation of small peptide fragments is an inevitable consequence of protein turnover and proteolytic processes occurring within tissues. From this perspective, many circulating dipeptides may simply represent incidental byproducts of increased proteolysis associated with tumor growth.

On the other hand, the existence of biologically active dipeptides demonstrates that short peptide sequences can possess functional properties that influence cellular physiology. Distinguishing between these possibilities is therefore essential for understanding the biological significance of circulating dipeptides observed in cancer metabolomics.

Addressing this question will require integrated studies combining metabolomics, peptide biochemistry, and functional assays to determine whether specific dipeptides exert measurable biological effects in cancer-related contexts. These examples highlight the diversity of dipeptides detected in biological systems and their potential functional relevance.

## 5. Dipeptide Signatures in Cancer Metabolomics

High-resolution metabolomics has enabled the comprehensive profiling of small molecules in biological samples, revealing complex metabolic alterations associated with cancer. Recent advances in high-resolution mass spectrometry and multi-omics integration have further improved the sensitivity, coverage, and reproducibility of metabolite detection in cancer studies, enabling more robust identification of low-abundance metabolites, including small peptides [67]. In addition to well-characterized metabolites such as amino acids, lipids, and central carbon intermediates, many metabolomic studies have reported the presence of short peptide fragments, including dipeptides, in plasma, serum, and tumor tissues [2,12].

Although these molecules are frequently detected across independent studies, their biological significance is rarely explored in detail. In many cases, dipeptides are reported as part of broader metabolomic signatures without mechanistic interpretation. Nevertheless, the repeated observation of specific dipeptides across multiple cancer types suggests that their presence may reflect underlying metabolic processes associated with tumor growth and host responses.

### 5.1. Dipeptides in Plasma and Serum Metabolomics

Several metabolomic analyses comparing plasma or serum samples from cancer patients and healthy individuals have identified alterations in circulating dipeptide levels [18,19,68]. Although only a limited number of studies have specifically reported dipeptides as primary analytes, accumulating metabolomic evidence indicates that dipeptides are frequently detected as part of broader metabolic signatures. The currently available studies are therefore presented as representative examples rather than an exhaustive list. Representative studies reporting specific dipeptides in cancer metabolomics are summarized in Table 4. These studies often employ untargeted metabolomics platforms capable of detecting a wide range of small molecules, including short peptide species.

Commonly reported dipeptides include combinations of branched-chain amino acids, such as leucine, valine, and isoleucine, as well as aromatic amino acids, including phenylalanine, tyrosine, and tryptophan. Examples include dipeptides such as Val–Leu, Leu–Leu, Tyr–Leu, and Phe–Phe, which have been detected in circulating metabolomic profiles in multiple studies.

The apparent enrichment of these residues in frequently detected dipeptides may reflect multiple factors. First, branched-chain and aromatic amino acids are relatively abundant in proteins, increasing the likelihood of their occurrence in proteolytic fragments [8,29]. Second, protease specificity and cleavage patterns can favor the generation of peptides containing hydrophobic or aromatic residues [35,36,37]. Finally, the physicochemical properties of such dipeptides, including hydrophobicity and ionization efficiency, may enhance their stability and detectability in mass spectrometry-based metabolomic platforms [52].

In many cases, the abundance of these dipeptides is increased in cancer patient samples relative to healthy controls. The precise origin of these changes remains unclear, but they are likely to reflect processes such as enhanced proteolysis, altered amino acid metabolism, or increased protein turnover associated with tumor progression. Most of these observations, however, come from untargeted metabolomic studies in which dipeptides are not the primary focus, and their identification is often based on putative annotation rather than confirmation with authentic standards. This limitation lowers confidence in the specificity and reproducibility of reported dipeptide changes across studies.

Despite differences in sample types, analytical platforms, and study design, several common patterns can still be recognized. Across studies, dipeptides are often detected as minor components within broader metabolomic signatures, and the number of reported dipeptides per study remains relatively limited.

In addition, most studies report relative changes in dipeptide abundance rather than absolute quantification, and consistency across independent cohorts remains modest. These limitations highlight the need for systematic and quantitative comparisons across studies to better define the role of circulating dipeptides in cancer metabolomics. Taken together, these findings suggest that currently reported dipeptide signatures are more reflective of global metabolic perturbations rather than specific tumor-derived signals. Without quantitative validation and mechanistic linkage, it remains difficult to determine whether individual dipeptides represent functionally relevant metabolites or indirect markers of increased proteolysis.

Collectively, these studies suggest that currently reported dipeptide signatures are more indicative of global metabolic disruption rather than specific tumor-derived signals. Without quantitative validation and mechanistic linkage, it remains difficult to determine whether individual dipeptides represent functionally relevant metabolites or indirect markers of increased proteolytic activity.

### 5.2. Dipeptides in Tumor Tissue Metabolomics

Dipeptides have also been detected in metabolomic analyses of tumor tissues. Studies analyzing metabolic profiles of resected tumors have reported the presence of multiple short peptide species that differ in abundance compared with adjacent normal tissues [70,71].

The accumulation of dipeptides within tumor tissues may arise from several factors. Tumor cells frequently exhibit elevated rates of protein synthesis and degradation, leading to increased intracellular peptide generation. In addition, enhanced autophagy and lysosomal degradation pathways can contribute to the production of peptide intermediates during protein recycling.

Tumor-associated protease activity within the extracellular matrix may further contribute to peptide generation within the tumor microenvironment. The resulting peptide fragments may remain within tumor tissues or enter the circulation through tumor-associated vasculature. Nevertheless, it remains difficult to determine whether these dipeptides represent functional metabolites or incidental products of increased protein degradation, as most studies do not include functional validation or flux analysis.

### 5.3. Recurrent Dipeptide Patterns Across Studies

While Section 5.1 and Section 5.2 summarize individual studies and observed dipeptide patterns, this section integrates these findings to highlight common trends and potential biological implications of circulating dipeptides in cancer. Although individual studies may report different peptide profiles depending on analytical platforms and sample types, several dipeptides appear repeatedly in cancer-associated metabolomic signatures [12]. For example, metabolomic profiling studies in neuroendocrine tumors have identified consistent alterations in circulating dipeptides, including Val–Leu, Thr–Ala, and Trp–Phe, suggesting that recurrent detection patterns may reflect shared metabolic perturbations rather than random fragmentation [5].

Many of these recurrent dipeptides contain amino acids that play important roles in cancer metabolism [20,22], including branched-chain and aromatic amino acids, which are central to metabolic adaptation and biosynthetic processes in proliferating cells. These amino acids are heavily involved in biosynthetic pathways, signaling networks, and metabolic adaptation processes that support tumor growth.

The repeated detection of similar dipeptide species raises the possibility that their presence may not be entirely random. Instead, they may reflect underlying metabolic processes such as selective protein degradation, preferential peptide stability, or regulated peptide transport.

However, systematic comparisons across studies remain limited, and the functional significance of recurrent dipeptide patterns remains largely unexplored.

Taken together, these findings suggest that circulating dipeptides may reflect integrated metabolic processes involving protein turnover, tissue-specific activity, and tumor-associated alterations, although their precise biological and clinical significance remains to be fully established. However, quantitative comparisons across studies remain limited, and reported dipeptide patterns should be interpreted with caution.

The recurrence of specific dipeptides across studies may therefore reflect shared biochemical constraints, such as amino acid abundance, protease specificity, and analytical detectability, rather than tumor-specific biological regulation. This distinction is critical for interpreting whether these patterns represent biologically meaningful signals or methodological convergence.

### 5.4. Potential Sources of Variability in Dipeptide Detection

The interpretation of dipeptide signatures in cancer metabolomics is influenced by multiple sources of variability across preanalytical, analytical, and post-analytical stages. Preanalytical factors, in particular, play an important role in shaping observed dipeptide profiles. Dipeptide levels can be affected by sample collection and handling, including factors such as time to centrifugation, temperature control, and delays before processing. Hemolysis may also introduce intracellular peptides into plasma samples, potentially complicating interpretation. Storage conditions, such as duration and temperature, along with repeated freeze–thaw cycles, can further affect peptide stability, leading to degradation or artificial variation in measured dipeptide levels. In addition, ongoing proteolytic activity during sample storage—particularly in the absence of protease inhibitors or under suboptimal temperature conditions—can lead to the artificial generation of dipeptides from larger proteins or peptides, thereby complicating their interpretation as endogenous circulating metabolites. These issues highlight the importance of standardized sample handling protocols in metabolomic studies, as such variability can introduce significant inter-sample differences and obscure biologically meaningful variation.

Analytical factors further contribute to variability in dipeptide detection. Differences in sample preparation and extraction protocols can influence peptide recovery, particularly for small and labile dipeptides. Chromatographic separation is essential for resolving structurally similar dipeptides, including sequence isomers, which may not be distinguishable based on mass spectrometry alone. In addition, variations in mass spectrometry platforms, including resolution, ionization methods, and fragmentation strategies, can significantly affect the detection and quantification of dipeptides [52,72]. These analytical differences can result in inconsistent detection, identification, and quantification of dipeptides across studies, further complicating cross-study comparisons and biomarker validation.

Post-analytical considerations also play a key role in data interpretation. In untargeted metabolomic studies, dipeptides are often annotated based on accurate mass and database matching, which may not distinguish between sequence isomers or structurally similar compounds. As a result, many reported dipeptides should be considered putatively identified unless confirmed using authentic standards. This issue is particularly relevant for dipeptides, where sequence isomers (e.g., Leu–Val vs. Val–Leu) share identical masses but may differ in chromatographic behavior and biological origin. Confirmation using reference standards, retention time matching, and targeted MS/MS validation is therefore essential to ensure accurate identification.

Furthermore, the lack of standardized workflows across studies complicates direct comparisons of reported dipeptide profiles. Orthogonal validation approaches, such as targeted quantitative assays or isotope-labeled standards, are often required to confirm both the identity and reproducibility of detected dipeptides. These methodological limitations should be carefully considered when interpreting reported dipeptide signatures and assessing their biological relevance [52].

Despite these challenges, the increasing reproducibility of metabolomic datasets across independent cohorts suggests that at least a subset of circulating dipeptides represent genuine biological metabolites rather than analytical artifacts.

Taken together, these preanalytical and analytical factors highlight that careful experimental design, standardized sample handling, and rigorous validation are essential for the reliable interpretation of circulating dipeptides in metabolomics-based biomarker studies. In practice, differences in analytical platforms have been shown to result in variable detection of small peptides across studies, even when analyzing similar biological samples, highlighting the need for standardized workflows and cross-platform validation [72].

### 5.5. Interpreting Dipeptide Signatures in Cancer

Taken together, the metabolomic evidence indicates that circulating dipeptides are consistently detectable in cancer-related metabolic profiles. However, the biological interpretation of these molecules remains ambiguous. Elevated dipeptide levels may reflect increased protein degradation, altered peptide metabolism, or systemic metabolic responses associated with tumor growth.

At the same time, the possibility that specific dipeptides possess biological activity cannot be excluded. If certain dipeptides participate in metabolic regulation, redox balance, or intercellular signaling, their detection in metabolomic studies may represent an underrecognized aspect of tumor biology.

Clarifying these possibilities requires integrating metabolomic observations with mechanistic studies aimed at understanding how dipeptides are generated, transported, and metabolized within cancer tissues and the host organism. For instance, targeted analyses of cancer tissues have demonstrated that specific dipeptides, such as Asp–Arg, exhibit consistent downregulation compared with normal tissues, supporting the notion that at least a subset of dipeptides may reflect regulated metabolic processes rather than nonspecific degradation [7].

## 6. Functional Hypotheses for Circulating Dipeptides in Cancer

Despite increasing detection in metabolomic studies, the functional roles of most circulating dipeptides remain incompletely understood. As illustrated in the conceptual framework (Figure 1), multiple biological mechanisms may contribute to the generation and potential functional roles of circulating dipeptides in cancer. At present, there is no unified framework explaining why specific dipeptides appear in circulating metabolic profiles of cancer patients. Several hypotheses can be considered to explain these observations, ranging from passive byproducts of protein degradation to potentially functional metabolites involved in tumor–host metabolic interactions [20,21,73]. These hypotheses are supported to varying degrees by experimental evidence. While some are grounded in established biochemical or physiological mechanisms, others remain largely speculative and are based on indirect observations or analogy with related metabolic processes. Importantly, most of these hypotheses are based on indirect evidence or analogy with related metabolic processes, and direct experimental validation of the proposed functions of circulating dipeptides remains limited.

Understanding the role of circulating dipeptides therefore requires consideration of multiple biological models that may operate simultaneously in cancer [74,75,76].

### 6.1. Dipeptides as Indicators of Enhanced Protein Turnover (Supported by Metabolomic and Biochemical Evidence)

One of the simplest explanations for circulating dipeptides in cancer is that they reflect metabolic byproducts of increased protein turnover. Tumor growth is associated with elevated rates of both protein synthesis and degradation, occurring within tumor cells as well as in surrounding tissues undergoing remodeling [6,21].

Enhanced proteolysis can generate a wide range of peptide fragments, which are further processed into smaller peptides and amino acids [7]. Under these conditions, some dipeptides may escape complete degradation and enter the systemic circulation, becoming detectable in metabolomic analyses. Consistent with this view, several studies have reported increased levels of multiple dipeptides in the plasma of cancer patients compared with healthy controls, suggesting a link between proteolytic activity and circulating peptide abundance [5,6,7].

From this perspective, circulating dipeptides may serve as indirect indicators of altered protein turnover associated with tumor growth, inflammation, and tissue remodeling. Similar mechanisms have been proposed to explain elevated levels of certain amino acids and peptide fragments observed in cancer-related metabolic profiles. The presence of endogenous dipeptides in mammalian tissues further supports the idea that at least some of these molecules arise from regulated metabolic processes rather than random degradation events [69].

### 6.2. Dipeptides as Metabolic Intermediates in Amino Acid Recycling (Supported by Transport and Metabolic Studies)

Another possibility is that circulating dipeptides represent intermediates within broader amino acid recycling pathways. In cells experiencing metabolic stress or high biosynthetic demand, protein degradation can provide an important source of amino acids for cellular metabolism.

Short peptides generated during proteolysis may serve as transient intermediates that facilitate amino acid recycling within tissues. Some of these peptides may be transported between cells or tissues through peptide transport systems before being hydrolyzed into free amino acids [16,47,77]. Experimental studies have demonstrated the functional expression of peptide transporters such as PEPT1 in cancer cells, supporting the possibility that dipeptides may participate in intercellular amino acid flux [30,41].

In this model, circulating dipeptides could reflect systemic amino acid flux associated with tumor metabolism. Their presence in blood plasma may therefore represent a snapshot of ongoing metabolic exchanges between tumor tissues and host metabolic compartments.

### 6.3. Dipeptides as Stress-Buffering Molecules (Supported by Experimental Evidence for Specific Dipeptides)

A third hypothesis is that certain dipeptides may function as protective molecules that help buffer metabolic stress. As discussed earlier, some dipeptides possess antioxidant or carbonyl-scavenging properties capable of neutralizing reactive molecules generated during oxidative stress [15,58]. In particular, histidine-containing dipeptides such as carnosine have been shown to exhibit antioxidant and carbonyl-scavenging activities, providing experimental support for a potential stress-buffering role.

Cancer metabolism is characterized by increased production of reactive oxygen species, metabolic intermediates, and electrophilic compounds that can damage cellular components. Dipeptides capable of reacting with these molecules may serve as small-molecule buffers that mitigate oxidative or carbonyl stress within tissues.

If such mechanisms operate in vivo, elevated levels of certain dipeptides in circulation may reflect adaptive responses to metabolic stress associated with tumor growth.

### 6.4. Dipeptides as Metabolic Signals (Largely Speculative)

An alternative possibility is that some circulating dipeptides may function as metabolic signals that influence cellular behavior. In recent years, numerous metabolites previously considered simple metabolic intermediates have been recognized as signaling molecules capable of regulating gene expression, immune responses, and metabolic pathways [20,75,78,79]. By analogy with these metabolite-derived signaling systems, it is conceivable that certain dipeptides may also possess signaling functions. Although direct evidence remains limited, small peptides such as kyotorphin (Tyr–Arg) have been reported to function as signaling molecules in biological systems, supporting the plausibility of peptide-mediated signaling mechanisms [54].

Although the signaling roles of endogenous dipeptides remain poorly characterized, their potential to interact with transporters, enzymes, or receptors further raises the possibility that they may participate in metabolic communication between tissues.

Within the tumor microenvironment, metabolites produced by tumor cells frequently influence immune cell activity, stromal cell behavior, and systemic host responses. If specific dipeptides possess signaling capabilities, they could contribute to previously unrecognized forms of metabolic communication within tumor–host interactions.

### 6.5. Tumor–Host Metabolic Communication (Conceptual/Integrative Hypothesis)

A final hypothesis integrates several of the mechanisms described above and considers circulating dipeptides as components of tumor–host metabolic communication. Tumor growth induces systemic metabolic changes affecting multiple organs, including liver, skeletal muscle, immune tissues, and the microbiome [45,74]. In this context, circulating dipeptides may contribute to metabolic interactions within the tumor microenvironment by influencing nutrient availability, redox balance, or intercellular communication.

Protein turnover and peptide metabolism occurring within these tissues could contribute to the circulating peptide pool. In turn, circulating peptides may influence metabolic pathways or signaling networks in distant tissues.

In this context, circulating dipeptides may represent metabolic fingerprints of tumor–host interactions rather than molecules produced exclusively by tumor cells. Understanding how these peptides are generated and distributed across tissues may therefore provide insight into systemic metabolic responses to cancer.

### 6.6. Distinguishing Functional Metabolites from Degradation Fragments

Ultimately, distinguishing whether circulating dipeptides represent functional metabolites or incidental degradation fragments remains a major challenge. Addressing this question will require integrated approaches combining metabolomics, peptide chemistry, transport biology, and functional cellular studies.

Key experimental strategies may include targeted metabolomic quantification of specific dipeptides, isotope tracing to determine their metabolic origin, and functional assays examining potential biological effects of candidate peptides.

Such studies may reveal that circulating dipeptides represent a heterogeneous group of molecules with diverse origins and functions. Some may simply reflect increased proteolysis, whereas others may participate in metabolic regulation or signaling pathways relevant to cancer biology.

Overall, these hypotheses likely represent a spectrum ranging from mechanisms supported by experimental evidence to more speculative conceptual models. Distinguishing between these categories will require further targeted experimental studies.

## 7. Clinical Implications and Biomarker Potential

The detection of circulating dipeptides in cancer metabolomic studies suggests that these molecules may have potential clinical relevance as biomarkers. Metabolomic profiling of blood samples is increasingly used to identify metabolic signatures associated with cancer diagnosis, prognosis, and treatment response [80,81]. Circulating metabolites are also being explored as minimally invasive biomarkers for cancer detection and monitoring, although their clinical application is still under investigation [76,82]. However, the diagnostic performance of circulating dipeptides, including sensitivity, specificity, and predictive value, has not been systematically evaluated. Most studies report relative changes in metabolite levels rather than quantitative performance metrics, which limits direct assessment of their clinical utility. Overall, current evidence suggests that circulating dipeptides are more likely to reflect systemic metabolic states rather than serve as standalone biomarkers. Their clinical value will likely depend on how well they can be integrated into multi-parameter models alongside other metabolites and clinical variables.

Short peptide metabolites may offer several potential advantages as biomarkers. Because dipeptides can arise from multiple biological processes—including protein turnover, tissue remodeling, and metabolic stress—they may provide integrated information reflecting both tumor activity and systemic host responses. This integrated metabolic perspective may complement traditional biomarkers that primarily reflect tumor-specific molecular alterations. At the same time, this lack of specificity may represent a limitation, as dipeptide levels can be influenced by multiple non-cancer-related factors, including diet, microbiome activity, and systemic protein turnover, potentially reducing their diagnostic specificity for cancer.

Several metabolomic studies have reported altered levels of specific dipeptides in plasma or serum samples from cancer patients compared with healthy individuals [5,18,19]. In some studies, these changes have been reported as measurable differences in relative abundance, with certain dipeptides showing consistent increases or decreases between cancer patients and controls, although absolute quantification and standardized validation remain limited. Although these findings are often embedded within broader metabolomic signatures, they suggest that circulating dipeptides may contribute to diagnostic or prognostic profiles.

Emerging experimental evidence further indicates that dipeptide-associated pathways may play active roles in tumor biology. For example, PEPT1-mediated dipeptide transport has been shown to promote tumor progression in hepatocellular carcinoma models by enhancing intracellular dipeptide availability and activating oncogenic signaling pathways [49]. These findings provide proof-of-concept that dipeptide-related metabolic processes may extend beyond passive byproducts of proteolysis and influence tumor behavior.

However, current evidence remains limited and context-dependent. Most studies rely on untargeted metabolomics without functional validation, and causal relationships between dipeptide levels and disease progression have not been systematically established. In addition, variability across analytical platforms and the lack of standardized validation frameworks complicate cross-study comparisons. Most available data are derived from cross-sectional analyses, and longitudinal validation in independent cohorts is required to determine reproducibility, disease specificity, and temporal dynamics.

The potential of circulating dipeptides as biomarkers of treatment response also remains largely unexplored. While systemic metabolic profiles are known to change following therapeutic interventions, including chemotherapy and targeted therapies [20,52,75], direct evidence linking specific dipeptide alterations to treatment outcomes is currently limited. Given their connection to protein turnover and metabolic activity, circulating dipeptides may reflect therapy-induced metabolic remodeling, but this hypothesis requires further validation in controlled clinical studies.

Despite these limitations, advances in high-resolution metabolomics and targeted peptide analysis are expected to facilitate more rigorous evaluation of circulating dipeptides as biomarkers. Translation into clinical practice will require standardized analytical workflows, well-defined reference ranges, and validation across diverse patient populations. In addition, careful consideration of confounding factors—such as diet, microbiome-derived metabolites, and baseline protein turnover—is essential to distinguish biologically meaningful signals from background variation. In parallel, dipeptide-related pathways may also have translational relevance in therapeutic delivery strategies, as peptide transport systems and dipeptide-based linkers are increasingly explored for targeted drug delivery in cancer treatment.

## 8. Conclusions and Future Perspectives

The growing use of metabolomics in cancer research has led to the detection of numerous small molecules whose biological roles are not yet fully understood. Among these, circulating dipeptides stand out as a relatively underexplored but potentially important class of metabolites.

Traditionally, dipeptides detected in biological samples have been regarded as incidental byproducts of protein degradation. However, this view may be overly simplistic, as accumulating evidence points to a more complex biological context. The existence of dedicated peptide transport systems, the biological activities observed for certain endogenous dipeptides, and the recurrent detection of specific dipeptide species in metabolomic studies all indicate that these molecules may have greater biological relevance than previously appreciated.

In the context of cancer, multiple processes—including enhanced proteolysis, autophagy, extracellular matrix remodeling, and systemic metabolic adaptation—may contribute to the generation and circulation of dipeptides. These mechanisms suggest that circulating peptide metabolites may reflect complex metabolic interactions between tumor tissues and the host organism.

At present, however, the functional roles of most circulating dipeptides remain unknown. Determining whether these molecules act as metabolic intermediates, stress-buffering compounds, signaling molecules, or simply degradation fragments represents an important challenge for future research.

Addressing these questions will require integrated experimental approaches combining metabolomics, isotope tracing, peptide biochemistry, and functional cellular assays. In particular, systematic identification of dipeptides that consistently appear in cancer-associated metabolic profiles may help prioritize candidates for mechanistic investigation.

As metabolomic technologies continue to improve, previously overlooked metabolite classes—including small peptides—may emerge as important components of metabolic regulation and tumor–host communication. A deeper understanding of circulating dipeptides may therefore reveal new aspects of cancer metabolism and potentially uncover novel metabolic biomarkers or therapeutic targets.

Circulating dipeptides may represent more than passive degradation products and could, in some contexts, reflect cancer-associated proteolytic remodeling, with potential relevance to signaling processes.

## Figures and Tables

**Table 1:** Biological sources and mechanistic features of circulating dipeptides in cancer. Multiple biological processes contribute to circulating dipeptides, with distinct proteolytic mechanisms and peptide characteristics depending on their origin.

Source	Core Mechanism	Peptide Features	Cancer Relevance	Key Reference
Intracellular proteolysis	Proteasomal degradation followed by cytosolic peptidase trimming	Short peptides with sequence-dependent C-terminal residues; further processed into dipeptides	Increased protein turnover in proliferating tumor cells	[29]
Autophagy and lysosomal degradation	Lysosomal proteolysis mediated by cathepsins	Heterogeneous peptide fragments generated under acidic conditions; further trimmed by peptidases	Upregulated under metabolic stress and nutrient limitation	[25]
Extracellular proteolysis	Protease-mediated ECM degradation (MMPs, cathepsins, serine proteases)	Extracellular peptide fragments subject to exopeptidase trimming	Associated with tumor invasion and microenvironment remodeling	[24]
Tumor cell death	Apoptotic caspase-mediated cleavage and necrotic cell lysis	Controlled intracellular fragmentation (apoptosis) vs. uncontrolled extracellular proteolysis (necrosis)	Enhanced during tumor progression and therapy-induced cell death	[38]
Host tissue catabolism	Systemic protein breakdown (e.g., muscle proteolysis)	Broad spectrum of peptide fragments reflecting systemic metabolic stress	Observed in cancer cachexia and systemic inflammation	[45]
Microbiome metabolism	Bacterial proteolysis of dietary and host proteins	Microbial-derived peptides with distinct sequence composition	Contributes to circulating metabolite diversity and host–microbiome interactions	[46]

**Table 2:** Representative dipeptides identified in metabolomic studies.

Dipeptide	Composition	Activity	Mechanism	Cancer Relevance	Reference
Carnosine	β-Ala–His	Antioxidant, antiglycation	Carbonyl scavenging	Anti-proliferative effects	[15]
Anserine	β-Ala–1-methylhistidine	Antioxidant	ROS buffering	Oxidative stress protection	[15]
Homocarnosine	GABA–His	Neuromodulatory	Neurotransmission modulation	Metabolic regulation	[15]
Tyr–Arg (Kyotorphin)	Tyr–Arg	Neuroactive	Signaling activity	Indicates peptide signaling potential	[54]
Pro–Hyp	Pro–Hydroxyproline	Collagen-derived	Tissue remodeling	ECM turnover	[55]
Gly–His	Gly–His	Metal chelation	Metal ion binding	Redox balance	[56]
Ala–His	Ala–His	Antioxidant	Radical scavenging	Stress response modulation	[57]

Abbreviations: Arg, arginine; Gly, glycine; His, histidine; Hyp, hydroxyproline; Pro, proline; Tyr, tyrosine. Note: Not all listed dipeptides are cancer-specific; some are included as representative circulating or biologically active dipeptides reported in metabolomic studies.

**Table 3:** Functional roles of dipeptides in cancer-related contexts.

Function	Dipeptides	Mechanism	Evidence	Cancer Relevance	Reference
Antioxidant/carbonyl scavenging	Carnosine, anserine	ROS and carbonyl scavenging (histidine-dependent)	Experimental (in vitro/in vivo)	Redox regulation	[15,26]
Metabolic modulation	Carnosine	Modulation of glycolysis and mitochondrial metabolism	Cell-based	Tumor energy metabolism	[27]
Amino acid supply	Various dipeptides	Hydrolysis to free amino acids by peptidases	Established	Supports biosynthesis	[10,31]
Transport-mediated uptake	Various dipeptides	PEPT1/PEPT2-mediated uptake (proton-coupled)	Physiological/cancer cell	Nutrient supply under stress	[16,30,47]
Signaling (putative)	Tyr–Arg (Kyotorphin), others	Potential receptor interaction (unclear mechanism)	Limited	Tumor–host communication	[54]
ECM-derived signaling	Pro–Hyp	Collagen-derived peptide signaling	Experimental	Microenvironment remodeling	[55]

Note: Evidence levels indicate the strength of experimental support. Some functional roles remain hypothetical and require further validation.

**Table 4:** Dipeptides reported in cancer metabolomic studies.

Dipeptide	Cancer Type	Sample	Direction	Reference
Val–Leu	NETs vs. control	Plasma	Increase	[5]
Thr–Ala	NETs vs. control	Plasma	Increase	[5]
Trp–Phe	NETs vs. control	Plasma	Increase	[5]
Thr–Gly	NETs vs. control	Plasma	Decrease	[5]
Ser–Ala	NETs vs. control	Plasma	Decrease	[5]
Ser–Val	NETs vs. control	Plasma	Decrease	[5]
Ser–Hydroxyproline	NETs vs. control	Plasma	Increase	[5]
HT + TH (His–Thr/Thr–His)	HCC (hepatitis vs. non-hepatitis)	Tumor	Decrease (hepatitis)	[6]
VI, IY, IE, TI, VN, VT	HCC (hepatitis vs. non-hepatitis)	Adjacent tissue	Increase (hepatitis)	[6]
Asp–Arg	Colorectal cancer vs. normal	Tissue	Decrease	[7]
Multiple dipeptides	Multi-tissue (non-cancer)	Tissue	Variable	[69]

Note: Direction indicates relative change compared with control samples. NETs, neuroendocrine tumors; HCC, hepatocellular carcinoma. The final entry represents non-cancer-specific profiling studies included to illustrate broader dipeptide distribution across tissues. This table presents representative examples rather than an exhaustive list.

## Data Availability

No new data were created or analyzed in this study. Data sharing is not applicable to this article.

## Abbreviations

ATP	Adenosine triphosphate
ECM	Extracellular matrix
HCC	Hepatocellular carcinoma
MMPs	Matrix metalloproteinases
MS	Mass spectrometry
NETs	Neuroendocrine tumors
PEPT1	Peptide transporter 1 (SLC15A1)
PEPT2	Peptide transporter 2 (SLC15A2)
ROS	Reactive oxygen species
SLC15	Solute carrier family 15 peptide transporters
SLC15A1	Solute carrier family 15 member 1
SLC15A2	Solute carrier family 15 member 2
UPLC	Ultra-performance liquid chromatography
